# Microphysiological Systems for Studying Cellular Crosstalk During the Neutrophil Response to Infection

**DOI:** 10.3389/fimmu.2021.661537

**Published:** 2021-04-27

**Authors:** Isaac M. Richardson, Christopher J. Calo, Laurel E. Hind

**Affiliations:** Department of Chemical and Biological Engineering, University of Colorado – Boulder, Boulder, CO, United States

**Keywords:** neutrophil, microfluidics, infection, *in vitro* models, innate immunity, cell-cell interactions, inflammation, antimicrobial functions

## Abstract

Neutrophils are the primary responders to infection, rapidly migrating to sites of inflammation and clearing pathogens through a variety of antimicrobial functions. This response is controlled by a complex network of signals produced by vascular cells, tissue resident cells, other immune cells, and the pathogen itself. Despite significant efforts to understand how these signals are integrated into the neutrophil response, we still do not have a complete picture of the mechanisms regulating this process. This is in part due to the inherent disadvantages of the most-used experimental systems: *in vitro* systems lack the complexity of the tissue microenvironment and animal models do not accurately capture the human immune response. Advanced microfluidic devices incorporating relevant tissue architectures, cell-cell interactions, and live pathogen sources have been developed to overcome these challenges. In this review, we will discuss the *in vitro* models currently being used to study the neutrophil response to infection, specifically in the context of cell-cell interactions, and provide an overview of their findings. We will also provide recommendations for the future direction of the field and what important aspects of the infectious microenvironment are missing from the current models.

## Introduction

The innate immune response to infection is a complicated process requiring a coordinated effort by many cell populations. Neutrophils are one of the first cells to arrive at an infection and are critical in limiting pathogen spread, but their response must be tightly regulated. Defects in neutrophil recruitment can lead to recurrent, unresolved infections and excessive neutrophil activity can lead to chronic inflammation and tissue damage; therefore, understanding the signals driving neutrophil recruitment is critical for controlling their response following an infection. It has been shown that the tissue environment and cellular interactions can have a significant impact on neutrophil function but new ways of studying the innate immune system are needed to determine how these interactions affect neutrophil function.

Two experimental systems are predominantly used for studying the innate immune response to infection: *in vitro* “cells-in-a-dish” and *in vivo* animal models. While animal models allow researchers to visualize the neutrophil response in a physiologically relevant environment that includes cellular interactions, their inherent complexity makes teasing apart the role of specific signals or interactions difficult. Therefore, *in vitro* models where primary human cells can be used are a necessary complement to animal models. Unfortunately, traditional *in vitro* systems lack important components of the infectious microenvironment including cell-cell interactions, three-dimensional structures, and a tissue mimic or extracellular matrix. Recently, several physiologically relevant *in vitro* models have been developed that incorporate these features. These studies have demonstrated the importance of including relevant geometries, cell-cell interactions, and cell matrix interactions in investigating the immune response.

## The Neutrophil Response to Inflammation

As the first cellular responders during inflammation, neutrophils serve as the foot soldiers of the innate immune response. Within minutes of infection or injury, neutrophils activate and migrate from the blood vessel to the site of inflammation where they clear pathogens and signal for the activation and recruitment of other immune cells. To achieve this rapid response, neutrophils employ non-specific antimicrobial activities, such as the generation of reactive oxygen species (ROS), that damage both pathogenic and host cells. Consequently, excessive neutrophil activity contributes to the pathology of several inflammatory diseases, including rheumatoid arthritis and gout (1, 2). Conversely, insufficient neutrophil activity is associated with recurrent and more severe infections as encountered by individuals with neutropenia, chronic granulomatous disease, and leukocyte adhesion deficiency (3–5). Therefore, it is crucial that neutrophil activity is highly regulated to prevent excessive collateral damage of healthy tissue, while still protecting the host from pathogens.

**Figure 1:**
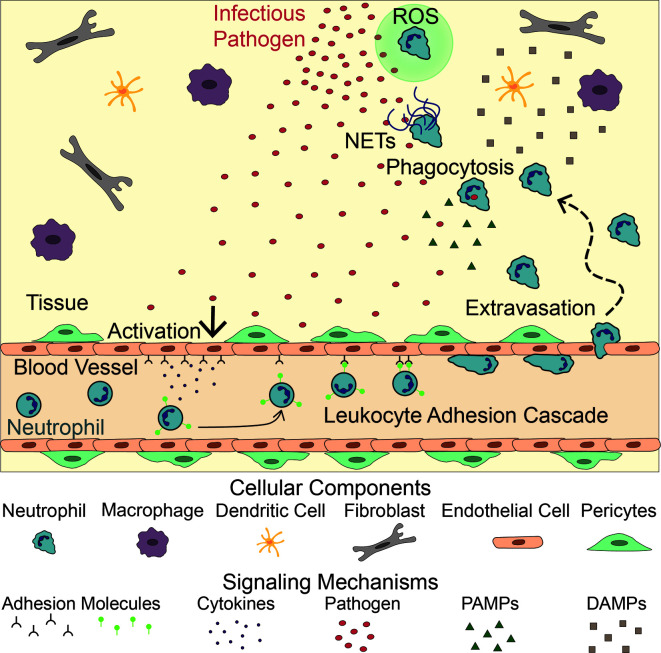
Neutrophil Response to Infection. Following infection, endothelial cells lining the vasculature become activated, releasing adhesion molecules and cytokines. These signals activate neutrophils, initiating the leukocyte adhesion cascade. Neutrophils then extravasate through the blood vessel and migrate to the site of infection following PAMPs, released by the pathogen, and DAMPs released by tissue resident cells (macrophages, dendritic cells, fibroblasts). There, they fight the infection by releasing NETs and Reactive Oxygen Species (ROS), and directly phagocytosing the pathogen.

Neutrophil recruitment to inflammation is mediated by an intricate meshwork of cellular interactions including, but not limited to, interactions between neutrophils, endothelial cells, other immune cells, and pathogens (6–10). Following infection, neutrophils escape the circulation through a series of interactions with endothelial cells, which line the blood vessel lumen, in a process known as the leukocyte adhesion cascade (**Figure 1**). Upon activation by signals from infected or injured tissue, blood vessel endothelial cells upregulate selectins, which then bind and capture circulating neutrophils (11, 12). These neutrophils then roll along the endothelium, accumulating bonds between integrins on their surface and adhesion molecules on the endothelial surface. Eventually, the neutrophils stop rolling and begin crawling along the endothelium, extending protrusions in search of a point to extravasate, or migrate, through the blood vessel (13, 14). Extravasation, whether transcellular (through an endothelial cell) or paracellular (between two endothelial cells), is mediated by the binding of adhesion molecules on the endothelial cell surface and their corresponding neutrophil ligands (14–18). After extravasation, neutrophils release neutrophil elastase to make a hole in the basement membrane of the blood vessel and migrate into the tissue (19). While this process is primarily governed by neutrophil-endothelial cell interactions, other cell types, such as monocytes, have been shown to influence neutrophil extravasation (20).

Once in the tissue, neutrophils follow chemoattractant gradients to locate and migrate to the site of inflammation. Neutrophils have over 30 receptors on their surface that recognize proinflammatory signals including cytokines released by tissue resident cells, damage-associated molecular patterns (DAMPs), and pathogen-associated molecular patterns (PAMPs) that guide their movement (21) (**Figure 1**). DNA, high mobility group protein B1, N-formyl peptides, extracellular matrix proteins, adenosine triphosphate (ATP), and uric acid are examples of DAMPs that drive early neutrophil recruitment in tissue. Their roles in this process have been previously reviewed (22–24). Tissue resident cells, such as macrophages and mast cells, also recognize DAMPs, PAMPs, and inflammatory cytokines. Upon stimulation, these resident cells produce and secrete inflammatory cytokines of their own, including interleukin-8 (IL-8), a potent neutrophil chemoattractant (25–28). As chemoattractants populate the local environment, they establish a gradient that directs neutrophils to the site of inflammation (29–32).

At the site of inflammation, neutrophils begin a neutrophil-recruitment feedback loop by releasing interleukin-1β (IL-1β), which activates macrophages, dendritic cells, γδ T cells, and endothelial cells to produce and release chemokines to recruit more neutrophils (33, 34). In addition to inflammatory cytokines, tissue resident cells and migrating neutrophils produce lipid mediators, most notably leukotriene B_4_ (LTB_4_), major contributors to a sustained neutrophil response (35–40). In both sterile and infected neutrophil responses, neutrophils swarm at the site of inflammation. Swarming is a process whereby neutrophils cluster around necrotic tissue and pathogens, including bacteria, fungi, and parasites (41). At the inflammatory site neutrophils continue to secrete LTB_4_ and express integrins to facilitate swarming. These swarms form a seal encased by late recruited macrophages and monocytes (42).

Upon reaching the site of inflammation, neutrophils employ various antimicrobial techniques to clear cellular debris as well as contain and kill pathogens. Their primary method of pathogen clearance is engulfment or phagocytosis (43, 44). Additionally, neutrophils attack pathogens by releasing ROS during respiratory bursts (45–47) and producing neutrophil extracellular traps (NETs) (48–50). NETs consist of decondensed neutrophil DNA and associated proteins in a web-like structure that prevent pathogens from spreading, while marking them for phagocytosis (48, 49).

As is the case with extravasation, neutrophil antimicrobial activity is influenced by interactions with other cells (50–52). Additionally, ROS and NETs contain proinflammatory signals that activate other cells, including neutrophils, to sustain and increase the inflammatory response. If not properly regulated, these signals can cause unwanted autoimmune responses and disease (53–56). Pathogens also influence neutrophil antimicrobial activity (57). For example, whether or not a neutrophil produces NETs is partially determined by the size of microbes (58).

Interaction with macrophages is especially important in the regulation of both neutrophil recruitment and resolution. Monocyte-derived macrophages clear NETs to prevent an overactive immune response, resolving inflammation (59). Dysregulation of this process, namely in the case of Lupus macrophages, leads to inflammasome activation in response to NETs, which in turn triggers the release of inflammatory cytokines inducing further NET production, or NETosis (60). Additionally, tissue resident macrophages downregulate neutrophil swarming and activity by hiding microlesions from neutrophils in tissue (61). Macrophages also inhibit sustained neutrophil recruitment and neutrophil-mediated killing in fungal infections by preventing fungal germination, a process that activates neutrophils (62). Finally, macrophages play a role in phagocytosis of neutrophils and their components, helping to resolve inflammation (63, 64).

To better understand the neutrophil response to infection, regulatory interactions between neutrophils and other cells, both host and pathogenic, must be studied in experimental systems that account for cell-cell interactions. While early studies with simple *in vitro* and complex *in vivo* models have gleaned fundamental knowledge of these interactions, they have key limitations. More recently, a range of physiologically relevant microfluidic devices have been developed to circumvent these limitations. These devices give a controlled, highly tailorable environment with low cost and high throughput for the study of human immune cell interactions. This review will focus on *in vitro* devices and how they have helped elucidate the effects of neutrophil interactions with host and pathogenic cells on the neutrophil response.

## *In Vitro* Models for Studying the Neutrophil Response

Historically, studies investigating the role of neutrophils in the immune response have been conducted in two types of models: simple *in vitro* systems, primarily investigating isolated interactions between two cell populations or a single cell population and an activating signal, and complex *in vivo* models, such as zebrafish or mice. These systems have led to important findings that serve as a knowledge base for the field; however, they have limitations. Simple *in vitro* models fail to capture the three-dimensional architecture of an *in vivo* environment along with pertinent physical cues. Furthermore, isolating cell types and investigating their interactions neglects important cellular signals from their environment. Conversely, animal models provide a complex and physiologically relevant three-dimensional environment to study immune responses; however, they are costly, low throughput, have a high degree of variability, and do not always translate to the human immune response. As such, recent efforts have focused on developing new experimental platforms for studying the neutrophil response to infection that include the physiological relevance of *in vivo* systems while preserving the advantages of studying cells *in vitro*.

**Figure 2:**
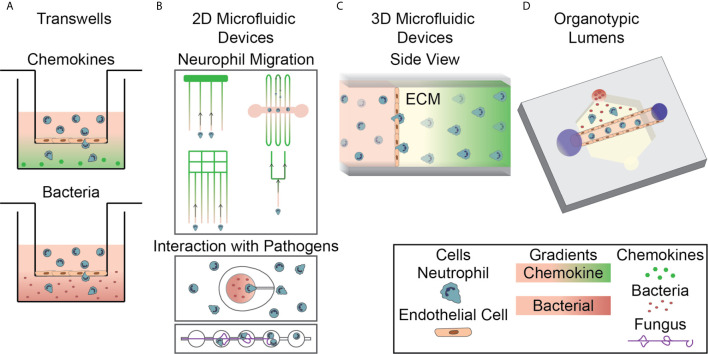
*In Vitro* Systems for Studying the Neutrophil Response to Infection. **(A)** Transwell assays, a well-in-well system with a porous membrane divider, are used to investigate neutrophil migration to chemokines (top, green gradient) and bacterial sources (bottom, red gradient) through cellular monolayers. **(B)** 2D microfluidic devices are used to investigate various aspects of neutrophil migration, including neutrophil reverse migration and migration through bifurcations, to soluble chemokines (top). Devices have also been designed to investigate direct interactions between neutrophils and both bacterial and fungal pathogens (bottom). **(C)** 3D microfluidic devices are used to investigate neutrophil migration to soluble chemokines in an extracellular matrix hydrogel following extravasation through an endothelium. Neutrophils migrate through an endothelial monolayer, seeded on the hydrogel, and into the ECM. **(D)** Organotypic microfluidic devices include a model vasculature containing endothelial cells in a relevant lumen geometry. These devices use both chemokines and live pathogens to induce migration.

Traditionally, simple *in vitro* models, such as Transwell assays and Dunn chambers, have been used to study neutrophils in two-dimensional environments. The first experiments investigating cell-cell interactions and the neutrophil response to infection used a simple Transwell assay. In general, these models consist of a well-in-well system in which endothelial monolayers are formed on porous membranes in the top well and inflammatory signals are added into the bottom well (**Figure 2A**). Neutrophils are then added to the top well and the number of neutrophils that migrate through the monolayer into the bottom well is quantified (65). This type of device has been used to model the innate immune response in different environments by altering the vascular cell sources and varying activation signals (66–77). For example, these devices have been used to study neutrophil migration in response to common inflammatory chemoattractants, including IL-8 and N-formylmethionine-leucyl-phenylalanine (fMLP) (29, 78) (**Figure 2A**, top), and in response to live bacterial infections, including *Escherichia coli*, *Streptococcus pneumoniae*, and *Staphylococcus aureus* (79) (**Figure 2A**, bottom). They have also been used to study the neutrophil response in the presence of additional supporting cell types such as pericytes (80, 81). Transwell assays are well designed for use in investigating overall neutrophil migratory behavior and their interaction with an endothelium, but, due to their design, they only allow for end-point analysis. Therefore, more complex models are required for real time monitoring of neutrophil behavior.

Animal models, primarily mice and zebrafish, have been the standard for conducting studies in complex, physiologically relevant systems (82, 83). Unlike Transwell assays, these animal models allow for *in vivo* imaging of neutrophils in a complex environment. In addition to live imaging of neutrophils, animal models have been used to identify complex neutrophil activity not seen in simple *in vitro* models, such as NETosis (48), migration away from a wound following interaction with a macrophage (84), and reverse transendothelial migration (85), the process whereby extravasated or partially extravasated neutrophils migrate back through the endothelium to renter the blood vessel. While these models allow for live imaging and can capture complex neutrophil phenomena, they do not always translate to human neutrophil activity. Furthermore, their inherent complexity makes it difficult to isolate and investigate the role of specific cell-cell interactions in regulating the neutrophil response. Consequently, recent work has focused on developing physiologically relevant *in vitro* microfluidic devices that combine the advantages of simple *in vitro* models while mimicking the *in vivo* environment.

Microfluidic devices are well-suited for studying the immune response because they can be designed with customizable geometries and require low reagent and cell volumes. These factors make them ideal for working with primary human cells and for precisely controlling the spatiotemporal presentation of signaling molecules and pathogens. During their response *in vivo*, neutrophils are exposed to activating signals from both tissue resident cells and the pathogen at different times and locations; therefore, spatiotemporal resolution of signal presentation is critical for modeling the *in vivo* environment and developing an understanding of how neutrophils respond to these varying signals. Additionally, they are straightforward to create and use, cost-effective, and can be multiplexed for high-throughput studies. These devices can be designed to include cellular components, tissue components, and architectures relevant for modeling the human immune system. Furthermore, they can be created to monitor innate immune cell function through many mechanisms including time-lapse imaging of cell behavior, collection and evaluation of cells, and quantification of soluble signals. Importantly, microfluidic devices allow for single-cell analysis which is of particular interest for studying neutrophils as recent reports have found high levels of neutrophil heterogeneity (86).Recently, researchers have developed increasingly complex devices incorporating physiologically relevant components and structures. These devices have brought about new insights into the role of both tissue architecture and neutrophil interactions with tissue components, vascular and stromal cells, and pathogens in modulating the innate immune response.

Several groups have developed microfluidic devices that allow for real time imaging and evaluation of the neutrophil response. These devices have been specifically designed to include elements that allow them to investigate individual neutrophil functions. Microfluidic devices have been designed for studying mechanisms of neutrophil migration (87–91), NETosis (92–94), ROS generation (92, 95) and, more recently, direct interactions between neutrophils and pathogens (**Figure 2B**). While many devices use representative chemokines to model an infectious source, devices have recently been designed to incorporate live, intact bacteria and fungi (**Figure 2B**, bottom). The addition of live pathogens increases the relevance of the neutrophil response and allows researchers to investigate the direct mechanisms of interaction between neutrophils and pathogens. A few recently described two-dimensional models for investigating neutrophil interactions with *Aspergillus fumigatus (A. fumigatus)* follow a similar design. The different models all have a chamber for loading of fungal conidia, space for outward hyphal growth to occur, and a separate loading port for the introduction of neutrophils. One device was designed to investigate the influence of directed migration on neutrophil interactions with the fungus *A. fumigatus* by requiring neutrophils to migrate up an fMLP gradient into a chemotaxis chamber before interacting with fungal spores (96). Devices have also been developed to investigate other neutrophil functions following interaction with fungal pathogens including one containing an array of fungal clusters designed to monitor neutrophil swarming (97). In addition to fungal pathogens, devices have been designed to investigate neutrophilic response to bacteria. A device of this nature was developed with ‘war theaters’ consisting of an inner microchamber seeded with bacteria inside a larger chamber seeded with neutrophils, allowing researchers to study the neutrophil recruitment and interactions with growing bacteria (**Figure 2B**, bottom) (98). These microfluidic devices have allowed researchers to investigate important components of the neutrophil response in an effective, high-throughput, and easily repeatable way and have led to critical discoveries about neutrophil-pathogen interactions that could only be studied in highly controlled environments. However, these devices do not account for key components of the *in vivo* infectious microenvironment, including three-dimensional structures, and do not replicate important events during the neutrophil response to infection such as extravasation through the vasculature. Therefore, devices with additional complexity have been developed to investigate the influence of those factors.

It has become apparent that multicellular interactions have a significant impact on the neutrophil response to infection. Additionally, migration in a three-dimensional system is not always well represented by migration in a two-dimensional model (87, 99, 100). As a result, microfluidic devices have been designed that incorporate multiple cell populations to investigate their interactions in three-dimensional environments. Interaction with endothelial cells is an important first step in neutrophil extravasation as part of their response to infection, therefore there is a particular interest in investigating neutrophil-endothelial cell interactions and many microfluidic devices have been designed to study how this interaction directs the neutrophil response (101–105). A consistent theme in the design of these devices is the creation of an endothelial cell layer on a gel surface, separating neutrophils from a chemoattractant chamber (**Figure 2C**). These models generally use a synthetic hydrogel or collagen as the surface for growing the endothelial monolayer. Neutrophil migration across this endothelial layer is then monitored using time-lapse microscopy and evaluated using end point analyses. The increased physiological relevance for investigating the neutrophil response in three-dimensions and the additional imaging mechanisms available for real-time study represent significant improvements over the Transwell system, however, these devices do not include relevant architectures and additional cell types.

Recent work has demonstrated the importance of incorporating relevant three-dimensional structure into microfluidic devices for studying the innate immune system. Specifically in the case of the endothelium, it was discovered that endothelial cells have different protein expression and secretion profiles when grown in a relevant lumen geometry compared to a two-dimensional monolayer (106). This altered endothelial cell signaling could have a direct effect on neutrophil migration and function. Several microfluidic models have been developed for investigating the vasculature (107–110) and recently these models have been expanded to look at migration of neutrophils out of the vasculature. Models of the vasculature for studying signaling of the endothelium in three-dimensional architectures for the purposes of angiogenesis or inflammatory environments have been reviewed elsewhere (111, 112).

As the importance of physiologically relevant architectures becomes more evident, more devices are being created that incorporate geometries that mimic the *in vivo* environment. The most relevant architecture to be considered when studying the innate immune response to infection is the blood vessel lumen; therefore, devices that incorporate model lumens are becoming increasingly more common (**Figure 2D**). In general, these devices are formed by creating a hollow structure in a hydrogel that is then seeded with endothelial cells to form a luminal monolayer. The specific strategies used for fabrication, activation, and incorporation of flow into these devices differ between model designs. An early high-throughput device design used viscous finger patterning to create continuous lumens in hydrogels within microchannels (113). This approach was further developed into the LumeNEXT system (114). LumeNEXT creates lumens by polymerizing an extracellular matrix (ECM) solution around a PDMS rod that when removed leaves behind a hollow lumen. This device has been used for studies investigating the role of live pathogens (115) and cell-cell interactions, including with endothelial cells (116, 117), during the neutrophil inflammatory response. The LumeNEXT system has also been used in combination with the Stacks open microfluidic system (118). The Stacks system uses hollow discs filled with ECM gels stacked atop one another to create a continuous matrix. This device, and others like it, allow for the formation of soluble gradients within the matrix. Cells migrate from the lumen to a chemoattractant source through these Stacks, which are then separated allowing distinct neutrophil subpopulations to be collected based on migratory ability (119). Others have used alternative methods for fabricating lumen structures. Endothelial-lined channels have been created using *in vivo* images of vascular networks as a pattern to create channels using polydimethylsiloxane (PDMS). These channels contain conduits to a tissue compartment, allowing neutrophils to migrate out of the endothelium (95). Introducing a model lumen into microfluidic devices for studying innate immunity has led to new insights and opened new research directions.

The field of tissue engineering has been investigating the vascularization of hydrogels for years and has developed innovative technologies for creating vascular networks. Systems using sacrificial polymer fibers or rods, similar to the LumeNEXT device are common (120, 121); however, more creative approaches have also been developed. Grigoryan et al. recently developed a particularly novel method for creating a complex, entangled vascular network using food dye additives as photoabsorbers for projection soft lithography. They then used this technology to create perfusable vascular networks that model lung alveolae (122). While these technologies have not extended to the investigation of the immune system, the potential for further development of microfluidic devices mimicking *in vivo* biology for the investigation of the neutrophil response could use these platforms for inspiration.

A majority of the previously discussed microfluidic devices require a neutrophil isolation step in which neutrophils removed from whole blood. This isolation allows for the investigation of neutrophils specific role in innate immunity; however, it also removes neutrophils from blood components that may influence neutrophil behavior and created a neutrophil environment that is not entirely representative of *in vivo* conditions. Additionally, the process of neutrophil isolation from whole blood can result in artificial activation of neutrophils, leading to inaccurate results. Therefore, researchers have developed microfluidic models that allow for the use of whole blood as the source of neutrophils, eliminating the neutrophil isolation step. By circumventing the isolation step these models avoid unintentional neutrophil activation and have higher throughput than models that require neutrophil purification from blood. In general, these devices contain a whole blood loading chamber connected by a channel to a chemotaxis chamber where chemoattractant is added and neutrophil migration can be evaluated (88, 123–127). Continued development of these devices will allow for further insights into signals controlling neutrophil function.

Microfluidic devices, especially those designed to use whole blood, have shown great potential for use in a variety of clinical applications. Devices allowing for the use of whole blood are particularly beneficial in clinical settings where their efficiency can have real time health implications. Additionally, these devices have a lower blood volume requirement than other device designs making them far less invasive. One such device, developed for the clinical diagnosis of asthma, isolates neutrophils from whole blood added to the system using a two-step process in which neutrophils bind to P-selectin then other blood cells are washed away. Asthma is then diagnosed using chemotaxis measurements (128). Devices have also been designed for the diagnosis of sepsis by measuring spontaneous neutrophil motility in blood (129, 130) and for monitoring NET prevalence within the blood post burn injury or sepsis (93). These types of whole blood microfluidic devices will continue to allow for quick and non-invasive diagnosis of a variety of innate immune related diseases.

## Neutrophil Interactions With the Blood Vessel

During an immune response, neutrophils must first extravasate through the blood vessel before navigating the extracellular matrix to reach the site of inflammation (**Figure 1**). This is a multi-step process governed by interactions between neutrophils and blood vessel cells, including endothelial cells, pericytes, smooth muscle cells, and fibroblasts. Endothelial cells line the lumen of the blood vessel and are the first cells neutrophils encounter during the inflammatory response; therefore, neutrophil-endothelial cell interactions have been studied most thoroughly. Less well studied are the interactions with cells lining the sub-luminal components of the blood vessel, such as pericytes, smooth muscle cells, and fibroblasts (**Table 1**).

**Table 1:** Summary of Results.

Cell Type	Model	Infectious Sources	Major Result	Reference
**Blood Vessel**				
• Endothelial Cells	• Transwell	• IL8, C5a, GM-CSF, fMLP, LPS	• Human neutrophil elastase, Rho, Rho kinase, plasminogen activator inhbitor-1, and ribosomal p70S6 kinase drive neutrophil TEM	(70, 71, 73, 76)
	• Transwell		• Mac-1 and LFA-1 on neutrophils and ICAM-1, ICAM-2, and PECAM-1 on endothelial cells mediate neutrophil TEM	(65, 67, 69, 72, 75)
	• 2D	• TNF-α Activated ECs	• Stiffer substrates enhance neutrophil TEM due to myosin-light chain dependent increases in endothelial cell contractility	(131–133)
	• 3D	• fMLP, IL8, LTB4	• fMLP is a more potent neutrophil chemoattractant than IL8, suggesting a hierarchy of pro-migratory signals	(101, 102, 105)
	• Organotypic (LumeNEXT)	• Bacteria (*P. aeruginosa*)	• Endothelial secretion of IL-6 and GM-CSF enhanced neutrophil migration and lifetime	(116)
	• Organotypic (LumeNEXT + STACKS)	• IL8	• TEM increases expression of genes for ROS production, cell adhesion, and chemokine receptors	(119)
	• Orgoanotypic (Bioinspired Vasculature)	• fMLP	• Neutrophils adhere to endothelial cells near bifurcations in a protein kinase Cδ- dependent manner	(134)
• Pericytes	• Transwell	• TNF-α Activated ECs/PCs	• Endothelial cell secretion of MIF decreased pericyte contractility and barrier function * via* reduced phospho-myosin light chain kinase	(135–137)
	• Transwell	• IL-17 Activated Pericytes	• Conditioned media from IL-17 activated pericytes increased neutrophil polarization, TNFα, IL1α, IL1β, IL8 secretion, phagocytosis	(81, 138)
• Fibroblasts	• Transwell	• RA, UV light, cystic fibrosis	• Inflammatory fibroblasts increased neutrophil adhesion to endothelial cells and TEM	(139, 140)
**Immune Cells**				
• Neutrophils	• 2D	• fMLP	• At bifurcations, leading neutrophils perturb the chemoattractant gradient, directing following neutrophils to take opposite path	(141)
			
	• Organotypic	• Fungus (*A. fumigatus*)	• LTB4 signaling between neutrophils leads to swarming	(117, 142–144)
• Monocytes	• Transwell	• Infected monocytes	• Neutrophils inhibit expression of IL6 and IL8 of infected monocytes	(145)
	• Organotypic (LumeNEXT)	• A. Fungus (*fumigatus*)	• Monocytes increase the neutrophil response in a MIP-1 and LPS dependent manner	(117, 146)
• Dendritic Cells	• Transwell	• Fungus (*A. fumigatus*)	• Neutrophil derived α-defensins induce DC migration and DC secretion of IL8 induces neutrophil migration	(147, 148)
• T Cells	• Transwell	• INFγ + LPS Activated Neutrophils	• Activated neutrophils secrete CCL2 and CCL20, inducing Th17 chemotaxis and activated Th17 cells secrete IL8, inducing neutrophil chemotaxis	(149)
**Pathogens**				
• Bacterium	• Transwell	• Bacteria (*Escherichia coli*) and LPS	• Neutrophils display a more potent response to live bacteria than to LPS	(79)
	• 2D	• Bacteria (*S. aureus*)	• Bacterial proliferation and neutrophil recruitments kinetics determine neutrophils’ ability to clear bacterial pathogens	(98)
	• Organotypic (LumeNEXT)	• Bacteria (*P. aeruginosa*)	• Activation of endothelial cells by *P. aeruginos*a led to increased neutrophil migration and lifetime	(116)
• Fungus	• 2D	• Fungus (*A. fumigatus*)	• Chemoattractant gradients prime neutrophils to block fungal germination, leading to *de novo* tip formation of new hyphae which is independent of NETosis and NADPH oxidase activity	(96, 150)
	• Organotypic (LumeNEXT)	• Fungus (*A. fumigatus*)	• Neutrophils respond to *A. fumigatus* and their response is enhanced by paracrine and autocrine signaling	(117)

### Neutrophil-Endothelial Cell Interactions

Early approaches to studying neutrophil-endothelial cell interactions employed Transwell assays and were instrumental in identifying key molecules that govern neutrophil-endothelial cell interactions during neutrophil transendothelial migration (TEM). They employed a variety of neutrophil chemoattractants produced by host cells, including IL-8, complement component 5a (C5a), and granulocyte-macrophage colony-stimulating factor (GM-CSF), as well as by pathogens, including fMLP and lipopolysaccharide (LPS) (65–77). These studies identified cytosolic and extracellular molecules that facilitate neutrophil TEM, including human neutrophil elastase (HNE) (70), endothelial Rho and Rho kinase (71), plasminogen activator inhibitor-1 (73), and ribosomal p70 S6 kinase (76). Other studies focused on neutrophil and endothelial cell surface proteins, such as macrophage-1 (Mac-1) and lymphocyte function-associated antigen-1 (LFA-1) on neutrophils and intercellular adhesion molecule-1 (ICAM-1), ICAM-2, and platelet-endothelial cell adhesion molecule-1 (PECAM-1) on endothelial cells, and their roles in neutrophil-endothelial cell interactions (65, 67, 69, 72, 75). Furthermore, a handful of experiments have used Transwell assays to study how viral infections influence neutrophil TEM (151–153). These studies found that Respiratory Syncytial Virus-infected epithelial cells and Cytomegalovirus-infected endothelial cells induce rapid neutrophil TEM (151, 152). Studies employing Transwells have identified important signaling pathways regulating neutrophil-endothelial cell interactions but only allow for end-point analysis and miss key aspects of the infectious environment, including variable presentation of soluble signals and mechanical signals.

To further elucidate factors affecting neutrophil-endothelial cell interactions, researchers have created more biologically accurate systems by designing *in vitro* devices that incorporate various characteristics of the *in vivo* environment not captured by Transwells. One such characteristic is the varying abluminal matrix stiffness found in differing tissues or disease states. A model using endothelial monolayers seeded on polyacrylamide hydrogels of varying stiffness showed neutrophil TEM increases on stiffer substrates (131, 132). This was accomplished by enhancing endothelial cell contractility through a myosin light chain-dependent pathway. Furthermore, this device highlighted the importance of myosin II mediated contractility and actin polymerization in neutrophils, for the speed and completion of TEM, respectively (133). Together these studies show that the physical environment affects individual cell types and subsequently alters their interactions with other cells during inflammation.

While this polyacrylamide model gives a tailorable substrate stiffness, it is restricted to chemokinetic (random migration) studies, not allowing for chemotactic (directed migration) responses. Furthermore, it does not allow for migration analysis of neutrophils in three-dimensional environments. This is significant as neutrophil integrin regulatory proteins differentially affect migration in two- and three-dimensional environments (87). Therefore, three-dimensional models allowing for analysis of neutrophil chemotaxis have been developed (**Figure 2C**) (101, 102, 105). Using these devices, researchers have been able to elucidate the relative potency of various chemoattractants. It was demonstrated that fMLP is a more potent chemoattractant than IL-8 during the early stages of neutrophil TEM (101). In agreement with these results, it was determined that, in the presence of competing chemoattractant gradients, neutrophils preferentially migrate towards fMLP over IL-8 (105). Interestingly, studies with that same device found no preferential neutrophil migration between competing gradients of LTB_4_ and fMLP. Together, these results suggest a hierarchy in pro-migratory signals in directing neutrophil migration.

The effect of an endothelium on the neutrophil response to infections in unique organ environments, such as the lung, has been studied using microfluidic models specifically designed to replicate the *in vivo* environment (103, 104). These models stack an air channel on top of a liquid channel with a porous membrane separating the two. The membrane is seeded with epithelial and endothelial cell monolayers on the top and bottom sides, respectively. Using these lung models, researchers have found that stimulating, infecting, or replicating disease states in the epithelial cell layer activates the endothelial cell layer, resulting in neutrophil recruitment, activation, and TEM (103, 104).

These devices all contain a two-dimensional endothelial cell monolayer and, therefore, do not capture the *in vivo* architecture of the endothelium. This is important to note as the three-dimensional geometry of the endothelium affects growth factor and cytokine secretion levels as well as phenotypic behavior of endothelial cells (106). For this reason, microfluidic devices have been developed to employ an endothelial lumen to more accurately capture the shape of blood vessels *in vivo* (**Figure 2D**) (114). One such device, LumeNEXT, was used to investigate the neutrophil response to the bacterial pathogen *Pseudomonas aeruginosa*. Interestingly, it was discovered that neutrophil lifetime and migration towards *P. aeruginosa* were significantly increased when an endothelium was present, compared to neutrophils migrating in the absence of an endothelium (116). These studies were extended by combining LumeNEXT with Stacks, as described above, to investigate the effect of transendothelial migration (TEM) on neutrophil function. It was discovered that TEM neutrophils upregulate genes for ROS production, cell adhesion, and chemokine receptors and produce higher levels of ROS in response to phorbol-12-myristate-13-acetate (PMA) stimulation compared to their non-TEM counterparts (119).

Models with relevant vascular architectures have also revealed how vessel structure alters neutrophil-endothelial cell interactions and neutrophil function. The Kiani Lab found that neutrophils preferentially adhere to activated endothelial cells near bifurcations in a protein kinase Cδ-dependent manner using a model vasculature, patterned to mimic an *in vivo* network (134). This result was particularly interesting as it was previously discovered, using a device with a tailorable bifurcations, that wider bifurcation angles facilitate increased neutrophil adhesion (154). Together, these results demonstrate the importance of vascular architecture and the consideration of both luminal structure and vascular bifurcations when studying neutrophil-endothelial cell interactions.

A wide range of *in vitro* models have been used to study neutrophil-endothelial cell interactions. Each system has its noted advantages and disadvantages, making them ideal for different types of studies. From identifying neutrophil chemoattractants and endothelial cell activators to elucidating the impacts of the physical environment on cell-cell interactions, these tools help us deepen our understanding of neutrophil-endothelial cell interactions during the inflammatory response.

### Neutrophil-Pericyte Interactions

After extravasation through the endothelium, neutrophils encounter pericytes in the basement membrane of the blood vessel. Pericytes serve to physically stabilize blood vessels, regulate blood flow, and aid in vascular development, maturation, remodeling, and permeability. These distinct pericyte roles have been previously reviewed (155). More recently, studies have investigated the role of pericytes in neutrophil extravasation and migration. The Gonzalez Lab has conducted extensive research on the effects of pericytes in neutrophil TEM using endothelial cell and pericyte monolayers in Transwell assays (80, 81, 135–138). Through a series of studies, they found that tumor necrosis factor-α (TNF-α) activation of endothelial cells and pericytes generates competing pro- and anti-inflammatory signals. Specifically, they showed neutrophils migrate through pericyte monolayers to a lesser extent than they do through endothelial cell monolayers and migrate through bilayers of the two cell types at intermediate levels (135). They then demonstrated that the observed intermediate migration is, in part, due to the disparate effects of TNF-α on endothelial cells and pericytes. TNF-α activation has a pro-inflammatory effect on endothelial cells, inducing the secretion of macrophage migration inhibitory factor (MIF), which has a pro-inflammatory effect on pericytes (136, 137). Conversely, TNF-α activation has an anti-inflammatory effect on pericytes, leading to a decrease in the inflammatory phenotype of endothelial cells as indicated by decreased neutrophil TEM across endothelial monolayers treated with supernatants from TNF-α treated pericytes (136). Furthermore, they found that seeding endothelial monolayers on pericyte-derived basement membrane decreased neutrophil adhesion and migration through the endothelium. Specifically, they found inflammatory stimuli led to fibronectin-rich, collagen-poor protein deposition as well as augmented fibronectin and laminin specific MMP production. Both these factors facilitated increased neutrophil migration (81, 138). Together, these results imply that endothelial cell-pericyte paracrine signaling is important in the regulation of neutrophil transmigration. They also suggest *in vitro* models that do not incorporate pericytes may miss relevant interactions affecting neutrophil migration.

While these studies demonstrated the importance of pericytes in neutrophil migration, they were carried out using Transwell assays which do not allow for real-time investigation of neutrophil interactions with pericytes, and more complex *in vitro* devices have not been used to investigate the intricacies of human neutrophil-pericyte interactions in relevant architectures. These studies indicate that endothelial-pericyte interactions likely play an important role in neutrophil TEM. Endothelial cell signaling is highly dependent on physical cues such as flow and monolayer architecture; therefore, important signaling pathways may not be captured in simple Transwell models. The interaction of pericytes with endothelial cells in a lumen geometry has been investigated in the context of tissue engineering. Alimperti et al. developed a bicellular device including endothelial lumens surrounded by attached pericytes, or human bone marrow stromal cells exhibiting mural cell characteristics similar to pericytes (156). They demonstrated that while RhoA activity increases vascular permeability, Rac1 and N-cadherin help maintain barrier function. Additionally, they found LPS, TNF-α, and thrombin increased vascular permeability and resulted in the detachment of the stromal cells from the endothelial lumen. The detachment of pericytes from the endothelium during inflammation is not captured by Transwell studies. As such, physiologically relevant *in vitro* models are needed to study the role of this phenomenon during inflammation. In the future, this model could be adapted to study the effect of pericytes on neutrophil migration in a relevant *in vitro* environment.

### Neutrophil-Smooth Muscle Cell Interactions

After extravasation through the basement membrane, neutrophils must navigate their way through smooth muscle cells. It has been shown that exosomes and nitric oxide, secreted by neutrophils in response to bacterial stimulators (LPS or fMLP), alter smooth muscle cell morphology and functionality (157, 158). Furthermore, it is known that smooth muscle cells secrete IL-8 in response to IL-17 stimulation (159). Despite evidence of neutrophil-smooth muscle cell interactions during inflammation, sophisticated *in vitro* devices emulating this part of the *in vivo* environment have yet to be developed to study the crosstalk between these cells. However, a lung airway-on-a-chip model for studying interactions between the epithelium and smooth muscle cells has been previously created (160). The design of this device could be used to generate a similar on-chip platform for studying neutrophil-smooth muscle cell interactions during inflammation.

### Neutrophil-Fibroblast Interactions

Fibroblasts are present in the outer most layer of blood vessels, the adventitia. Fibroblasts are the main producers of ECM proteins and thus are crucial in tissue regeneration (161). However, they also play significant roles in inflammatory responses, as reviewed previously (162, 163). Early studies used simple culturing techniques to demonstrate fibroblasts’ ability to produce IL-8 (164). These culturing studies then advanced to separate neutrophils and fibroblasts with a Transwell filter coated with endothelial cells (165). These studies showed synovial fibroblasts from patients with rheumatoid arthritis increased the number of neutrophils adhering to endothelial cells compared to their healthy counterparts. Recently, a device incorporating a flat endothelium, fibroblasts in a sub-luminal collagen gel, and a keratinocyte monolayer was developed (139). Using this model, Kwak et al. demonstrated ultraviolet light-induced cytokine secretion by the resident cells increased neutrophil TEM. Mejías et al. developed a system for studying neutrophil recruitment from the vascular network incorporating flat epithelial and endothelial monolayers in addition to fibroblasts (140). By replacing the healthy epithelium with cystic fibrosis human bronchial epitheliums, they were able to induce a disease state that resulted in neutrophil recruitment. These sophisticated devices should serve as models for introducing additional cell types and reconfiguring the geometries to make even more physiologically relevant systems in the future.

Neutrophil-endothelial cell interactions have been studied thoroughly, in both simple and complex *in vitro* systems. However, evidence suggests that other vascular and stromal cells, including pericytes, smooth muscle cells, and fibroblasts impact neutrophil inflammatory responses. While some microfluidic devices have been created to incorporate subsets of these cells in one system, future work is needed to make more biologically accurate replications of blood vessels and the surrounding tissue that contain all relevant cells populations.

## Neutrophil Interactions With Immune Cells

**Figure 3:**
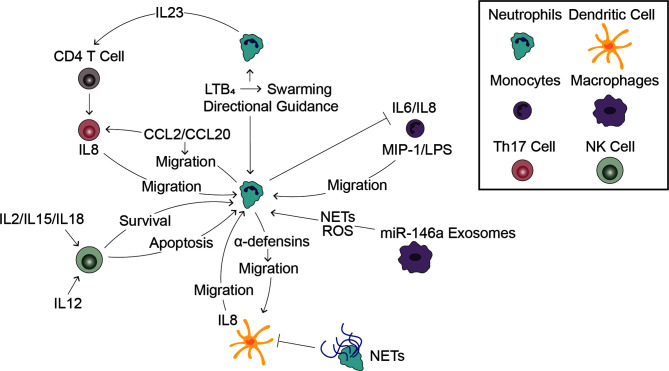
Interactions with Immune Cells Influences the Neutrophil Response. Cell-cell signaling between neutrophils and other immune cells plays a significant role in the innate immune response to infection. Leading neutrophils influence swarming and the directional migration of trailing neutrophils (top middle). Monocytes induce neutrophil migration and in turn, neutrophils inhibit pro-inflammatory signaling by monocytes (top right). Release of miR-146a rich exosomes induces neutrophil extracellular trap formation and reactive oxygen species generation (bottom right). Dendritic cells stimulate neutrophil migration while neutrophils have a dual effect on dendritic cells, stimulating migration through release of α-defensins while reducing DC production of inflammatory signals through signaling through NETs (bottom middle). NK cells can both promote neutrophil survival following stimulation by pro-inflammatory cytokines and promote neutrophil apoptosis following stimulation with anti-inflammatory cytokines (bottom left). Neutrophils stimulate Th17 cell migration and induce CD4 T cells to become Th17 cells, which in turn stimulate neutrophil migration (top left).

Inside of blood vessels, neutrophils communicate with other immune cells to efficiently navigate the vascular network and rapidly activate during an inflammatory response. Once outside of the blood vessel, neutrophils must maneuver through the ECM to reach the site of inflammation. Neutrophils achieve this by following various chemoattractant gradients generated by both pathogens and other host cells, including other immune cells (**Figure 1**). Furthermore, immune cells help activate and inactivate neutrophils as is necessary for inducing, sustaining, and eventually terminating inflammation (**Figure 3**).

### Neutrophil-Neutrophil Interactions

Neutrophils signal to other neutrophils during inflammatory responses, generally through a signal amplification mechanism. They are the primary source of LTB_4_, a proinflammatory lipid mediator, which is critical for the neutrophil response to bacterial, fungal, and parasitic infections (38, 117, 166). At sites of infection, neutrophil production of LTB_4_ has been shown to be important for swarming, a phenomenon by which neutrophils surround a pathogen and encase it to prevent it from spreading throughout the body (97, 167). Interestingly, the swarming response orchestrated by LTB_4_ varies in magnitude depending on the pathogen type, indicating a role for the pathogen as well in modulating this neutrophil function (97). While neutrophil swarming has primarily been studied *in vivo*, physiologically relevant microfluidic models of the neutrophil response have also shown LTB_4_-mediated neutrophil swarming in response to *A. fumigatus* (117, 142–144).

In addition to amplifying chemotactic signals through secondary gradients of LTB_4_, neutrophils communicate inside blood vessels by altering the physical environment. The vasculature is a complex network, and neutrophils must be able to navigate this network without forming “traffic jams.” To investigate how neutrophils avoid build ups, Wang et al. developed a field-goal shaped device where the channels create a “T” then continue up from both branches, simulating a vascular bifurcation (141). By controlling the width of the branch channels and the distance between neutrophils approaching the decision point, they determined leading neutrophils perturb the chemoattractant gradient and, to a lesser extent, the pressure gradient in the branch they traverse, causing closely trailing neutrophils to enter the opposite branch. Whether inside a blood vessel prior to activation or in the extracellular matrix during an inflammatory response, neutrophils are constantly communicating with one another to ensure they can respond rapidly and efficiently when the innate immune response is initiated.

### Neutrophil-Monocyte/Macrophage/Dendritic Cell Interactions

In addition to neutrophils, monocytes extravasate through the blood vessel and into the surrounding tissue following an inflammatory insult. Once in the tissue, they differentiate into either macrophages or dendritic cells, in response to the surrounding environmental cues (168, 169). Monocytes in each of these differentiation states have been shown to interact with neutrophils. Through a co-culturing method where monocytes and neutrophils were either allowed to have direct contact with one another or were separated by a Transwell filter, Tang et al. demonstrated neutrophils inhibit the expression of IL-6 and IL-8 by rhinovirus infected monocytes, reducing inflammatory signal production (145). Furthermore, using the LumeNEXT device, it was discovered that monocytes increase the neutrophil migratory response to *A. fumigatus* infection through a mechanism involving MIP-1 and to LPS in an extracellular nucleotide-dependent manner (117, 146).

Complex, three-dimensional microfluidic devices have yet to be designed to study neutrophil-macrophage interactions. However, simple *in vitro* approaches have been used to study the crosstalk between these cell types. It has also been shown oxidized low-density lipoproteins induce secretion of miR-146a rich exosomes by macrophages, which generate oxidative stress in neutrophils, increasing neutrophil ROS production and NET formation (170). Additionally, in a co-culturing study, neutrophils and macrophages were shown to cooperate in a contact dependent manner to eliminate macrophages infected with *Leishmania braziliensis* (171).

Like neutrophil-macrophage interactions, neutrophil-dendritic cell interactions have not been studied in complex devices mimicking the *in vivo* environment, but simple *in vitro* studies have been conducted. Transwell assays have been used to show α-defensins, proinflammatory peptides derived from neutrophils, induce immature dendritic cell migration and *A. fumigatus*-infected dendritic cells induce neutrophil migration through secretion of IL-8 (147, 148). Furthermore, incubating dendritic cells with NETs prior to LPS exposure was found to attenuate upregulation of dendritic cell markers and secretion of inflammatory cytokines (172).

Dendritic cells and macrophages play a crucial role in the both the innate and adaptive immune responses, but we have only begun to understand how interactions with these cells guide the neutrophil response. Current studies primarily use simple Transwell assays that do not allow for a detailed evaluation of neutrophil activation or function following interaction with monocytic cells; therefore, these studies should be expanded to complex devices that allow for real-time analysis.

### Neutrophil-Platelet Interactions

Platelets are non-nucleated, membrane bound packets of cytoplasm that are released into the blood by megakaryocytes residing in the bone marrow. These cell fragments predominantly serve to maintain blood vessel integrity as well as initiate and participate in clotting. Prior to extravasation, neutrophils interact with platelets in the blood. Most work investigating neutrophil-platelet interactions has focused on their interplay during thrombosis. Transwell studies showed that platelet factor 4, which is secreted by activated platelets, serves as a chemoattractant for neutrophils (173). Once drawn to the platelets, neutrophils adhere to platelets through selectin-ligand binding (P-selectin on platelets and L-selectin on neutrophils), as was demonstrated in parallel plate flow chambers (174). Flow chambers have also been used to show that platelets indirectly affect neutrophils by releasing platelet-derived extracellular vesicles (PEVs) that transiently bind to either endothelial cells or neutrophils. Upon binding PEVs induced increased adhesion molecule (ICAM-1 and VCAM-1) expression by endothelial cells and integrin (CD11b) expression on neutrophils, which promotes increased neutrophil-endothelial cell adhesion interactions (175). Collectively, these results indicate that platelets play a regulatory role in neutrophil transendothelial migration, yet their contribution to the neutrophil response to infection has not been studied in depth. Further investigation into the role of platelets in the neutrophil response should be conducted in multicellular systems that include endothelial cells, better recapitulating the *in vivo* environment.

### Neutrophil-Other Immune Cell Interactions

Of the remaining neutrophil-immune cell interactions, most *in vitro* work has been conducted with Transwell assays or by coculture of cells with and without porous membrane dividers. Neutrophil interactions with T cells, mast cells, and natural killer cells (NK cells) have been studied using these approaches. Neutrophil-T cell Transwell assays have revealed neutrophils induce Th17 chemotaxis by secreting CCL2 and CCL20 and activated Th17 cells secreted IL-8 for the recruitment of neutrophils (149). Furthermore, Toll-like Receptor 8 (TLR8) activated neutrophils have been demonstrated to secrete IL-23, which induces naïve CD4 T cells to become Th17 cells (176). Numerous mast cell studies have shown stimulation with various pathogens or pathogen-derived molecules induces secretion of inflammatory cytokines and chemokines that augment neutrophil migration (28, 177–179). Lastly, neutrophil-NK cell studies have revealed both contact dependent and independent crosstalk occur between the cells leading to changes in neutrophil receptor expression and survival. NK cells stimulated with proinflammatory cytokines, including IL-2, IL-15, and IL-18, send neutrophil survival signals, and increase expression of Fc γ receptor I (CD64), CD11b, and CD69 on neutrophils (180). Conversely, NK cells can also induce apoptosis of neutrophils in a caspase and direct contact dependent manner in response to IL-12, an anti-inflammatory cytokine (181). NK cells can also bring about neutrophil apoptosis following ROS production through binding of the MIC-A protein on neutrophils with the NKG2D protein on NK cells (182).

Neutrophils signal to and receive signals from numerous immune cell types that guide and regulate their activity during an inflammatory response. This cellular crosstalk has been investigated primarily in animal models and simple *in vitro* studies, primarily co-cultures of neutrophils with another cell type. In the same way Transwell inserts were used to discover key signaling molecules and interactions between neutrophils and endothelial cells or pericytes, co-culture studies have identified molecules immune cells use to signal to neutrophils. However, more complex microfluidic devices are needed to capture the nuances of these cell-cell interactions in more physiologically relevant environments.

## Neutrophil Interactions With Pathogens

Cellular signaling between a pathogen source and responding neutrophils plays a key and obvious role in directing the overall innate immune response. Pathogens release several signals driving the neutrophil response. Initially, pathogen derived peptides activate endothelial cells lining the blood vessel initiating the neutrophil extravasation process described previously. Once neutrophils have left the blood vessel, they reach the site of infection by following PAMPs and pathogen derived peptides released by the pathogen source (**Figure 1**). Recent work has shown the neutrophil response is dependent on the specific pathogen causing the inflammation (79). This suggests the innate immune response is tuned to respond specifically to distinct bacteria or fungi infecting the body. In responding to a pathogen source, neutrophils have multiple tools in their arsenal to use in attacking and slowing down the spread of the infection, including phagocytosis, swarming, ROS generation, and NETosis. Just as cellular interactions direct the migratory response of neutrophils towards the pathogen source, they also modulate the use of these antimicrobial response tools. Microfluidic devices have been created to investigate the specific and unique role the pathogen source plays in both directing neutrophil migration and modulating neutrophil effector functions upon interaction with the pathogen itself.

### Neutrophil-Bacterium Interactions

Neutrophil-pathogen interactions have been investigated since neutrophils were first discovered in the 19^th^ century. Early modern studies investigated the interactions between bacteria and neutrophils by introducing both cell types into solution together and monitoring the results (183). These studies primarily focused on the metabolic pathways involved during the interaction and the effects on respiration. As the field has evolved, the research focus has shifted to investigating the specific signaling events between the pathogen and neutrophils. The methods used for investigating these interactions have developed with improved experimental models. An early model design used a simple Transwell assay where neutrophils were seeded in the upper chamber and live, intact bacteria were seeded in the lower chamber to investigate if different pathogens distinctly directed the neutrophil response to infection (79). Significantly, this paper demonstrated neutrophils display a more potent response to the live, intact bacteria than to isolated bacterial peptides. Specifically, they found neutrophil migration towards *Escherichia coli* occurred at a rate ten-fold greater than towards LPS. While many current and past studies solely use bacterial peptides as their model for infection (66–77), this paper highlights the importance of using whole bacteria. Microfluidic devices of increasing physiological relevance have been designed to further investigate neutrophil-pathogen interactions. One such device used ‘war theatres’ (**Figure 2B**, bottom) to identify that bacterial proliferation and neutrophil recruitment kinetics were important factors in determining pathogen infection outcomes, as measured by neutrophils’ ability to neutralize bacteria (98). Advanced microfluidic devices incorporating physiologically relevant architectures and additional cellular components have also been designed to investigate neutrophil-pathogen interactions. It was discovered using an organotypic lumen model that activation of an endothelial lumen by the bacterial pathogen *Pseudomonas aeruginosa* led to an enhanced neutrophil response due to increased endothelial secretion of IL-6 and GM-CSF (116). This paper investigated neutrophil recruitment to a single bacterial source, but additional research is needed to further understand migration towards other types of bacterial pathogens.

### Neutrophil-Fungus Interactions

In addition to studying interactions between bacteria and neutrophils, several groups have investigated interactions between fungi and neutrophils, with many studies focusing on the environmental fungus *A. fumigatus*. *A. fumigatus* is a common opportunistic pathogen that, while harmless to most people, can cause life-threatening disease in immunocompromised individuals (184). It is well-studied, making it a good model pathogen for studying innate immunity in microfluidic devices. A series of studies using devices specifically designed for examining neutrophil interactions with *A. fumigatus* show neutrophils limit *A. fumigatus* growth through a variety of mechanisms. Jones et al. found an introduced chemoattractant gradient primed neutrophils to migrate to *A. fumigatus* conidia and block fungal germination and growth (96). Interestingly, this blockage in hyphal growth is counteracted by the fungus *via de novo* tip formation and development of a new hyphae near the interaction site. This fungal behavior was discovered and found to be independent of both NADPH oxidase activity and NETosis using a microfluidic device allowing for single cell analysis of fungal-neutrophil interactions (**Figure 2B**, bottom) (150). In the presence of high numbers of neutrophils, this leaves the hyphae vulnerable, but without a significant neutrophil response it can lead to aggressive *A. fumigatus* invasion, potentially describing a mechanism by which neutrophils protect against invasive *A. fumigatus* infections. Finally, studies have used unique microfluidic platforms to investigate molecules with therapeutic potential. One such study describes bifunctional compounds that bind to both the microbial target (*A. fumigatus*) and the neutrophil chemoattractant receptors. These compounds were able to assist neutrophils in slowing hyphal growth and were also able to enhance phagocytosis of conidia (185).

Neutrophil interactions with fungal infections have also been investigated in three-dimensional models containing more physiologically relevant components and architectures. The ability of neutrophils to migrate in three dimensions in these models is an improvement over previous two-dimensional devices as migration in a three-dimensional environment is not always well represented by migration in a two-dimensional model (87, 99, 100) Importantly, these models include endothelial cells which play an important role in modulating the innate immune response. *In vivo* studies have demonstrated a role for endothelial cells in altering the activation state of neutrophils and altering their response to infection (186). Therefore, it is critical to include these physiologically relevant architectures and components to elucidate an accurate understanding of neutrophil-pathogen interactions. Several three-dimensional microfluidic devices have been created with thematically similar designs but slight variations between them. In general, these devices consist of an endothelial cell coated lumen with neutrophils seeded within and a fungal source outside (**Figure 2D**) (115). Studies using three-dimensional models have found paracrine signaling, from monocytes through MIP-1 and other neutrophils through LTB_4_, plays an important role in driving the neutrophil response towards a source of *A. fumigatus* (117). These devices have provided new insights into the neutrophil response to fungal infections and identified paracrine signaling mechanisms influencing this response; however, *in vivo* studies point to an important role for other cell types, including macrophages, in regulating the neutrophil response to fungal infections. New devices containing additional relevant cell types are needed to fully understand the neutrophil response to infection.

Not only have microfluidic devices been designed to investigate interactions between neutrophils and different pathogen types; they have also been designed for investigating how neutrophils use different effector functions to combat these pathogens. In general, these devices involve neutrophils being captured, either by P-selectin (92) or by micropost arrays (93), and stained to visualize NET formation and ROS production under different conditions. One result of interest showed ROS production was necessary for neutrophils to form NETs (92). Studies using these devices have also shown an increase in NETs circulating in the blood following a major burn (93). Neutrophil use of effector functions is tightly regulated by cell-cell interactions.

Pathogens play an important signaling role in modulating neutrophil migration and effector functions as demonstrated by the papers discussed above. A common theme among papers investigating neutrophil-pathogen interactions is the diversity of neutrophil responses arising from different pathogens. This points to a critical need to better understand the uniqueness in each neutrophil-pathogen interaction and what drives these differences. The devices highlighted in this section have already played an important role in expanding the depth of understanding of this important relationship, but more work must be done to further our understanding of how cell-cell interactions contribute to the complicated signaling networks driving the neutrophil response. Specifically, the interaction of neutrophils with other tissue cells must be included in the analysis of neutrophils’ response to pathogens. It is known that interaction with vascular and other immune cells alters the activation state of neutrophils *in vitro* (116) and *in vivo* (85, 187); therefore, analysis of neutrophil interactions with pathogens in systems that do not include supporting cell populations give an incomplete picture of neutrophil activation and function.

## Conclusion

A properly regulated neutrophil response is critical for fighting infection while maintaining tissue homeostasis. Following infection, neutrophils must process a complex milieu of signals emanating from the cellular and physical components of their environment into an efficient and directed response. There has been a substantial effort to understand how the various signals neutrophils encounter drive their response using both *in vivo* and *in vitro* models. Numerous signaling pathways and cell-cell interactions have been identified as critical regulators of the neutrophil response using these models, but we still do not have a clear picture of how these signals are integrated into a single response following an infection. Our lack of understanding is derived from a few key limitations of current experimental systems. Animal models provide a complete overview of an immune response but do not always correlate to human disease and most current *in vitro* models lack at least one key component of an infectious microenvironment: a live source of infecting pathogen, relevant cell populations, and relevant architectures. To create a full picture of the signaling networks driving the neutrophil response, we must strive to develop new models, inspired by *in vivo* biology, that capture all relevant aspects of the infectious microenvironment. Initially, studies must be conducted to increase our understanding of how interactions with different cell types (smooth muscle cells, fibroblasts, macrophages, dendritic cells, NK and T cells) alter neutrophil function and activation using simple *in vitro* devices that allow for real-time analysis. To date, these studies have primarily been conducted using Transwell assays which prevent investigation into the morphologies of interacting cells, the modes of interaction, and the kinetics of neutrophil activation and function. These studies will incrementally increase our understanding of neutrophil function in the infectious microenvironment. Future studies should then focus on the development of devices that can include multiple cell populations known to influence the neutrophil response to infection, including macrophages, dendritic cells, pericytes, and stromal cells to investigate the complex cellular crosstalk occurring in an environment that most closely recapitulates tissues *in vivo*. This will require significant design optimization as different cell populations require diverse culture times, nutrients, and conditions *in vitro*; therefore, the authors suggest an iterative process in which single cell populations are included in each new design. In the design of these devices, special attention should be paid to integrate cellular populations that are most likely involved in multicellular signaling cascades that influence neutrophil migration using results from simple *in vitro* studies and *in vivo* work as a guide. Finally, evidence suggests that live pathogens provide a more relevant stimulus than peptides, yet many studies investigating neutrophil function still rely on the introduction of inflammatory peptides or single attractants. Therefore, there should be a significant emphasis in the field to use a variety of live pathogens to simulate infections rather than relying on bacterial derived peptides. By understanding how cell-cell interactions regulate the neutrophil response to infection, we can attempt to manipulate the response through the intelligent development of new therapeutics to treat infection.

## Author Contributions

IR, CC, and LH wrote and edited the manuscript. All authors contributed to the article and approved the submitted version.

## Conflict of Interest

The authors declare that the research was conducted in the absence of any commercial or financial relationships that could be construed as a potential conflict of interest.
